# Balancing selectivity vs stability using molecular dynamics and umbrella sampling

**DOI:** 10.1186/1758-2946-6-S1-O22

**Published:** 2014-03-11

**Authors:** Jeremie Mortier, Elisabeth K Nyakatura, Markus Miettinen, Carsten Baldauf, Gerhard Wolber, Beate Koksch

**Affiliations:** 1 Department of Biology, Chemistry and Pharmacy, Freie Universität Berlin, Takustraße 3, 14195 Berlin, Germany; 2 Department of Physics, Freie Universität Berlin, Takustraße 3, 14195 Berlin, Germany; 3 Fritz Haber Institute, Faradayweg 4-6, 14195 Berlin, Germany

## 

Coiled coils are highly represented in biologically relevant macromolecules involved in important biological functions, such as gene expression regulation. The coiled coil environment has the great advantage to provide two very well defined intermolecular recognition surfaces. The peptide system VPE-VPK is a rationally designed heterodimeric coiled coil structure [1,2]. The characteristic structure of the α-helical coiled coil allows randomizing the interaction partners of this dimeric system. Using a pool of VPE mutants that contains every possible combinations of the 20 canonical amino acids, specific binders could be searched empirically [1,3]. In this work, three key positions in the hydrophobic core were randomized in a VPE phage displayed library (Figure 1).

**Figure 1:**
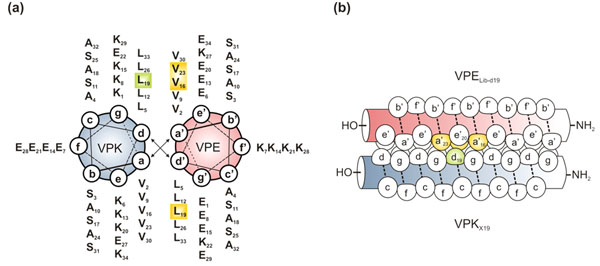
VPE-VPK represented as (a) a helical wheel diagram and (b) a ribbon diagram, with randomized positions in yellow, and the directly interacting position in green.

This screen led to the identification of a novel core packing between VPE and VPK. One single consensus sequence was selected by the system, bearing a tyrosine in the hydrophobic core. Surprisingly, the dimer selected by phage display has a lower stability compared to the mother system. This important result raises the central question of selectivity vs stability. In order to address both aspects, theoretical investigations were conducted using molecular dynamics within the Gomacs suite. Pulling apart the two helices up to 3.00 nm from each other, potentials of mean force were calculated by umbrella sampling with a view to compare the energy barriers of the mother dimer to the phage display variant.

## References

[B1] Nyakatura EK Reimann O Vagt T Salwiczek M Koksch B Accommodating fluorinated amino acids in a helical peptide environment Rsc Adv 2013 3 6319 6322 10.1039/c3ra41110a

[B2] Salwiczek M Samsonov S Vagt T Nyakatura E Fleige E Numata J Position-dependent effects of fluorinated amino acids on the hydrophobic core formation of a heterodimeric coiled coil Chemistry 2009 15 7628 7636 10.1002/chem.200802136 19579235

[B3] Vagt T Nyakatura E Salwiczek M Jäckel C Koksch B Towards identifying preferred interaction partners of fluorinated amino acids within the hydrophobic environment of a dimeric coiled coil peptide Organic & biomolecular chemistry 2010 8 1382 1386 10.1039/b917205j 20204211

